# Intra-Arterial Stem Cell Transplantation in Experimental Stroke in Rats: Real-Time MR Visualization of Transplanted Cells Starting With Their First Pass Through the Brain With Regard to the Therapeutic Action

**DOI:** 10.3389/fnins.2021.641970

**Published:** 2021-03-02

**Authors:** Daria D. Namestnikova, Ilya L. Gubskiy, Veronica A. Revkova, Kirill K. Sukhinich, Pavel A. Melnikov, Anna N. Gabashvili, Elvira A. Cherkashova, Daniil A. Vishnevskiy, Victoria V. Kurilo, Veronica V. Burunova, Alevtina S. Semkina, Maxim A. Abakumov, Leonid V. Gubsky, Vladimir P. Chekhonin, Jan-Eric Ahlfors, Vladimir P. Baklaushev, Konstantin N. Yarygin

**Affiliations:** ^1^Department of Neurology, Neurosurgery and Medical Genetics, Pirogov Russian National Research Medical University of the Ministry of Healthcare of Russian Federation, Moscow, Russia; ^2^Radiology and Clinical Physiology Scientific Research Center, Federal Center of Brain Research and Neurotechnologies of the Federal Medical Biological Agency of Russian Federation, Moscow, Russia; ^3^Cell Technology Laboratory, Federal Research and Clinical Center of Specialized Medical Care and Medical Technologies of the Federal Medical Biological Agency of Russian Federation, Moscow, Russia; ^4^Laboratory of Problems of Regeneration, Koltzov Institute of Developmental Biology of the Russian Academy of Sciences, Moscow, Russia; ^5^Department of Fundamental and Applied Neurobiology, Serbsky Federal Medical Research Centre of Psychiatry and Narcology of the Ministry of Healthcare of Russian Federation, Moscow, Russia; ^6^Laboratory of Biomedical Nanomaterials, National University of Science and Technology “MISIS”, Moscow, Russia; ^7^Laboratory of Cell Biology, Orekhovich Institute of Biomedical Chemistry of the Russian Academy of Sciences, Moscow, Russia; ^8^New World Laboratories, Laval, QC, Canada; ^9^Russian Medical Academy of Continuous Professional Education of the Ministry of Healthcare of the Russian Federation, Moscow, Russia

**Keywords:** real-time MRI, directly reprogrammed neural precursor cells, cell reprogramming, mesenchymal stem cells, intra-arterial cell transplantation, middle cerebral artery occlusion, experimental stroke

## Abstract

Cell therapy is an emerging approach to stroke treatment with a potential to limit brain damage and enhance its restoration after the acute phase of the disease. In this study we tested directly reprogrammed neural precursor cells (drNPC) derived from adult human bone marrow cells in the rat middle cerebral artery occlusion (MCAO) model of acute ischemic stroke using human placenta mesenchymal stem cells (pMSC) as a positive control with previously confirmed efficacy. Cells were infused into the ipsilateral (right) internal carotid artery of male Wistar rats 24 h after MCAO. The main goal of this work was to evaluate real-time distribution and subsequent homing of transplanted cells in the brain. This was achieved by performing intra-arterial infusion directly inside the MRI scanner and allowed transplanted cells tracing starting from their first pass through the brain vessels. Immediately after transplantation, cells were observed in the periphery of the infarct zone and in the brain stem, 15 min later small numbers of cells could be discovered deep in the infarct core and in the contralateral hemisphere, where drNPC were seen earlier and in greater numbers than pMSC. Transplanted cells in both groups could no longer be detected in the rat brain 48–72 h after infusion. Histological and histochemical analysis demonstrated that both the drNPC and pMSC were localized inside blood vessels in close contact with the vascular wall. No passage of labeled cells through the blood brain barrier was observed. Additionally, the therapeutic effects of drNPC and pMSC were compared. Both drNPC and pMSC induced substantial attenuation of neurological deficits evaluated at the 7th and 14th day after transplantation using the modified neurological severity score (mNSS). Some of the effects of drNPC and pMSC, such as the influence on the infarct volume and the survival rate of animals, differed. The results suggest a paracrine mechanism of the positive therapeutic effects of IA drNPC and pMSC infusion, potentially enhanced by the cell-cell interactions. Our data also indicate that the long-term homing of transplanted cells in the brain is not necessary for the brain’s functional recovery.

## Introduction

Ischemic stroke is one of the prevailing causes of disability and death throughout the world (Thrift et al., 2017; Skvortsova et al., 2018). The immediate therapeutic objective for acute ischemic stroke patients is prompt and efficient restoration of blood flow to ischemic brain tissue achieved by reperfusion therapy involving thrombolysis with intravenous tissue plasminogen activator or mechanical thromboextraction (Bhaskar et al., 2018; Powers et al., 2019). Both methods have limitations and are effective only if applied during a narrow “therapeutic window” after stroke onset, which is 4.5–9 h for systemic thrombolysis (Campbell et al., 2019) and no more than 24 h for mechanical thromboextraction (Albers et al., 2018; Nogueira et al., 2018). Moreover, even in the case of timely reperfusion therapy followed by the currently available neurorehabilitation, many patients suffer from life-long neurological deficits. It has been hypothesized that efficient ischemic stroke post-reperfusion treatment should include neuroprotection, control of neuroinflammation and autoimmune reactions, restoration of microcirculation and blood-brain barrier integrity, and enhancement of brain plasticity. Cell therapy is an emerging strategy with a potential to meet most of these requirements, as it has shown efficacy in brain restoration after the acute phase of stroke in animal models and showed promise in the I-II phase clinical trials (Diamandis and Borlongan, 2015; Wei et al., 2017). Studies are ongoing to select the cells most convenient for transplantation, and to determine the exact mechanisms of their therapeutic effects, the best transplantation time and route, and other conditions to enable high efficacy and safety of cell therapy in stroke. Concerning the choice of cell type, neural precursor cells (NPC) and mesenchymal stem cells (MSC).

NPC are innate ancestors of specific neuron types and demonstrate tri-lineage (neurons, astrocytes, oligodendrocytes) differentiation capacity *in vitro* (for review see Ottoboni et al., 2020). Therefore, they are obvious substitutes for the recipient’s neurons and glial cells eradicated or impaired by stroke. Indeed, NPC showed curative potential in animal stroke models and clinical tests (Chen et al., 2016; Stonesifer et al., 2017) and the ability to home into the allogenic or even xenogenic recipient’s brain and differentiate into mature neurons (Chu et al., 2003; Mine et al., 2013; Eckert et al., 2015). However, replacement of damaged cells is not the one and only or even the predominant mechanism of their therapeutic activity. In animal stroke models, transplanted allogeneic NPC most likely influenced brain neural, glial and immune cells and enhanced brain tissue protection and regeneration via secretion of cytokines, growth factors and other biologically active substances (Bacigaluppi et al., 2009; Baker et al., 2019; Vonderwalde et al., 2020). Since isolation of native human NPC directly from fetal or adult brain is associated with serious technical, legal and ethical problems, alternative methods of their production for preclinical studies and clinical needs have been developed and currently human NPC can be generated by neurogenic differentiation of embryonic stem cells (ESC) or induced pluripotent stem cells (iPSC) or by direct reprogramming of somatic cells. Each of those methods has advantages and disadvantages, but in terms of safety direct reprogramming is probably the best as it bypasses the pluripotent state. Cell products derived from pluripotent cells can display teratogenicity or undesired differentiation products if some of the cells skip differentiation and retain pluripotency. In addition, iPSC can acquire genetic and epigenetic abnormalities resulting in tumorigenicity and immunogenicity of their differentiated progeny (Yu et al., 2013; Nori et al., 2015). Direct reprogramming is likely to deliver less risky cell products. In this study we used directly reprogrammed NPC (drNPC) derived from human bone marrow mesenchymal cells (Ahlfors et al., 2019). These cells or their oligodendrogenic derivatives proved to be therapeutically effective in rodent (Nagoshi et al., 2008; Nori et al., 2018) and non-human primate (Baklaushev et al., 2019) models of spinal cord injury and in a mouse focal ischemic stroke model with formation of small cortical infarctions (Vonderwalde et al., 2020). In this study drNPC were transplanted into rats intra-arterially 24 h after the transient middle cerebral artery occlusion (MCAO). The rodent MCAO model of ischemic stroke first introduced by Koizumi et al. (1986) and modified to its present form by Longa et al. (1989). This model has been chosen as it has good reproducibility, large zone of ischemic lesion and is generally acknowledged as the one most closely mimicking human ischemic stroke (Fluri et al., 2015). Recently, our group advanced the model by introducing magnetic resonance imaging (MRI) control during operation, which allows to improving the success rate of procedure (Gubskiy et al., 2017). DrNPC were transplanted intra-arterially to provide targeted delivery to the ischemic lesion and to bypass filtering organs (lungs, liver, spleen) (Walczak et al., 2017).

MSC, a heterogeneous group of cells morphologically resembling fibroblasts and displaying an array of features favoring their use in cell therapy, can be easily isolated from several body tissues. MSC have been reported to possess remarkable immunomodulatory/anti-inflammatory (Yarygin et al., 2016; Weiss and Dahlke, 2019), neurotrophic (Eckert et al., 2013) and neurogenic (Dharmasaroja, 2009) properties. Insignificant immunogenicity (Ryan et al., 2005), teratogenicity, and tumorigenic activity (Hatzistergos et al., 2011) have made human MSC one of the most convenient candidates for the development of cell therapies. In the present study MSC was chosen as a positive control with known efficacy in stroke (Wang et al., 2018; Levy et al., 2019) and also as a cell type of mesodermal lineage, like the bone marrow cells that drNPC are derived from.

This work was mainly aimed to study the distribution, as well as homing within the brain of drNPC and pMSC after intra-arterial transplantation in MCAO rats. We consider the study of cell fate to be one of the key factors on the way to understand the mechanisms by which transplanted stem/progenitor cells exert their positive effects. Among all imaging modalities for stem/progenitor cells tracking in live animals (von der Haar et al., 2015; Bulte and Daldrup-Link, 2018), MRI combined with the labeling of cells with superparamagnetic iron oxide micro- and nanoparticles is one of the most convenient and relevant. In recent decades, this method has been widely used in basic and clinical studies (Bulte, 2009; Namestnikova et al., 2017b; Sehl et al., 2020). However, in the majority of investigations labeled cells were detected only after the completing of transplantation. For precise cell visualization in this study we used real-time MRI starting at the very onset of cell infusion, that allowed us to trace transplanted drNPC and pMSC starting from their first passage through the cerebral circulation. Real-time MRI for stem cell tracking was proposed recently by Walczak et al. (2017) and used in current work with modification according to our goals. Additionally, therapeutic efficacy of drNPC and MSC transplantation was assessed by evaluating post-stroke neurological deficits, calculating the survival rate, and MRI-based estimation of the infarct volume and frequency of hemorrhagic transformations.

## Materials and Methods

### Ethics Statement

The human biological material was obtained from the Perinatal Center of Kama Children’s Medical Center (KCMC) of Naberezhnye Chelny after getting informed consent from a donor. The study was approved by the local Ethical Committee of the Pirogov Russian National Research Medical University (Protocol No. 140 from December 15, 2014). All animal studies were approved by the local Ethical Committee of the Pirogov Russian National Research Medical University (Protocol No. 140 from December 15, 2014) and of Federal Research and Clinical Center of Specialized Medical Care and Medical Technologies (protocol No. 1_6_2019 from April 9, 2019). Experiments were carried out in accordance with directive 2010/63/EU on the protection of animals used for scientific purposes of the European Parliament and the Council of European Union dated September 22, 2010. All efforts were made to minimize the number of animals and exclude pain and other unpleasant effects for the animals. There were no restrictions in access to water and food (rodent chow), or other limitations. Animal studies are reported according to ARRIVE guidelines. The studies of cell transplantation into human participants were not conducted.

### Directly Reprogrammed Human Neural Precursor Cells

**FIGURE 1:**
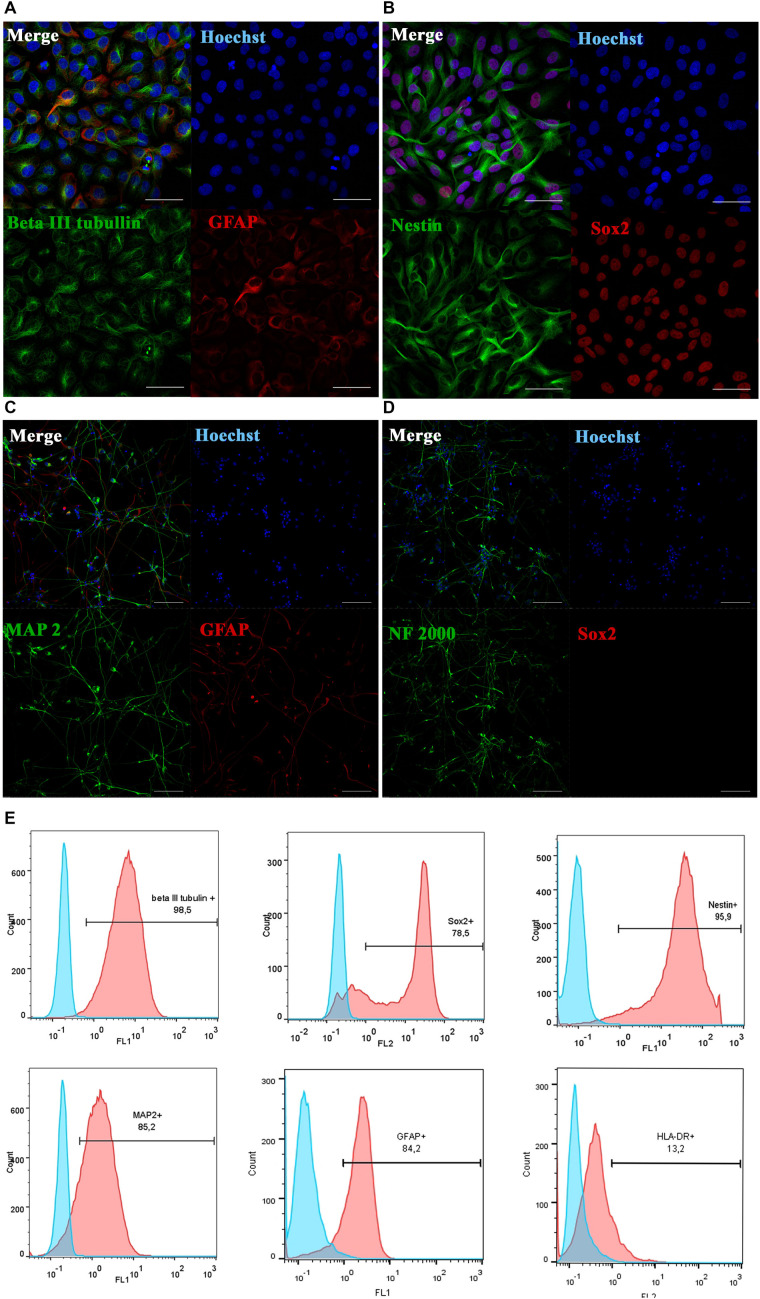
Phenotypic characterization and differentiation of drNPC. Undifferentiated drNPC stained positive for βIII-tubulin and GFAP **(A)**, Nestin and SOX2 **(B)**. Placing drNPC in the neuron-differntiation medium resulted in upregulation of mature neuronal markers MAP2 **(C)** and Neurofilaments NF2000 **(D)**, and the loss of SOX2 expression **(D)**. Scale bar = 50 μm in panels **(A,B)** and 100 μm in panels **(C,D)**. **(E)** Flow cytometry analysis of undifferentiated drNPC revealed the expression of βIII-tubulin+, Nestin+, SOX2+, GFAP+, MAP2b+ and lack of HLA-DR- expression. Blue graphs represent negative control (isotypic immunoglobulins); pink graphs – experimental samples with specific antibodies.

Directly reprogrammed neural precursor cells (drNPC) were kindly provided by New World Laboratories, Inc. (Laval, Quebec, Canada). They were prepared from cultured adult bone marrow MSC by direct reprogramming, skipping the pluripotency stage in the programming of human bone marrow MSC using transient transfection with the following 3 factors: Musashi−1 (Msi1), Neurogenin−2 (Ngn2), and methyl−CpG binding domain protein 2 (MBD2) (Ahlfors et al., 2019). Cells were cultured in Petri dishes (SPL Life Sciences, South Korea) coated with human laminin (L2020, Sigma-Aldrich), at 37°C in the atmosphere of 5% CO_2_ and 5% O_2_ in complete NeuroCult^TM^-XF Proliferation medium (StemCell Technologies) supplemented with B27 (1×, Gibco), 20 ng/ml of epidermal growth factor (EGF, Biolegend), 40 ng/ml of fibroblast growth factor-2 (FGF-2, Biolegend) and 100 ng/ml heparin (Stem cells technology). Cells were fed by replacing 50% of the medium every 36 h, passaged when they reached ∼80% confluence and after 5–7 passages prepared for transplantation in the following way: drNPC were dissociated using Accutase^®^ (StemCell Technologies, Canada), Accutase^®^ was inactivated with 100% knock out serum replacement (Gibco, United States) and the obtained cell suspension centrifuged at 250 × *g* for 4 min. Cells were washed twice in Dulbecco’s Phosphate-Buffered Saline (DPBS) and counted. A dose of 7 × 10^5^ cells in 2 ml of saline was used for each transplantation. Cell phenotype was checked by immunocytochemistry and flow cytometry (Figure 1). The absence of Mycoplasma was confirmed by PCR.

### Human Placenta-Derived Mesenchymal Stem Cells (pMSCs)

**FIGURE 2:**
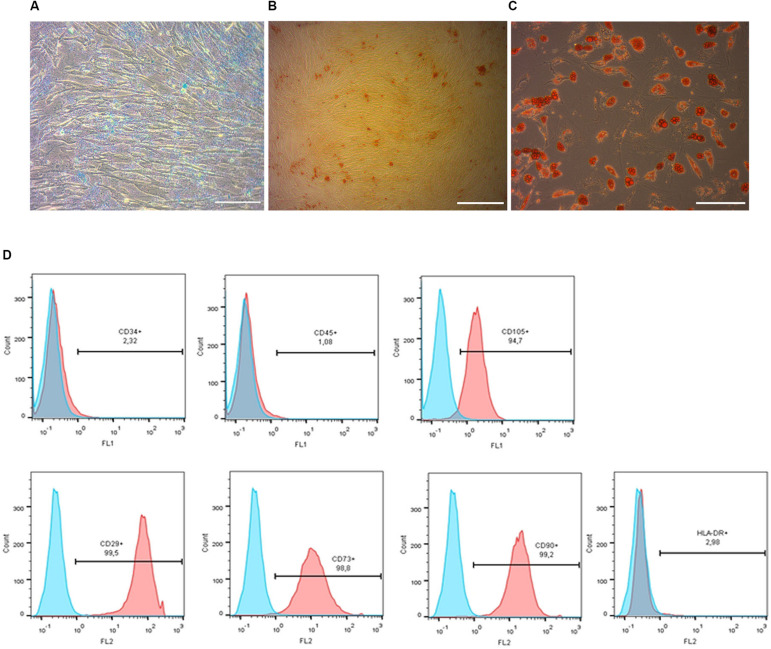
Phenotypic characterization and differentiation of pMSC. Chondrogenic **(A)**, osteogenic **(B)** and adipogenic **(C)** differentiation of pMSC, revealed by Alcian Blue, Alizarin Red and Oil Red staining, respectively. Scale bar = 100 μm. Flow cytometry analysis of the pMSCs’ surface antigen expression **(D).** Most cultured cells were positive for surface antigens typically expressed by mesenchymal stem cells: CD105+, CD29+, CD73+, CD90+ and negative for non-mesenchymal CD34- and CD45- markers. Blue graphs represent negative control (isotypic immunoglobulins); pink graphs – experimental samples.

Mesenchymal stem cells were isolated from human placenta collected after normal delivery at 40 weeks of gestation. The biological material was obtained from the Perinatal Center of Kama Children’s Medical Center (KCMC) of Naberezhnye Chelny using a conventional procedure described previously (Burunova et al., 2010) after getting informed consent from mothers. In brief, human placenta tissue samples were thoroughly minced with scissors, extensively washed with cold Hank’s solution and after gentle mechanical agitation incubated for 2 h at 37°C in Hank’s solution in the presence of 0.1% type I collagenase (Gibco). Collagenase was then inactivated with 10% FBS and tissue suspension centrifuged at 400 × *g* for 4 min at 25°C. Supernatant was discarded and pellet resuspended in the complete culture medium comprising DMEM-F12, 2 mM L-glutamine, 100 U/ml penicillin, 0.1 mg/ml streptomycin and 10% fetal bovine serum (all reagents from Gibco). The resuspended cell pellet was placed in T75 culture flasks and maintained in a humidified atmosphere under standard conditions (37°C, 5% CO_2_). Cells were allowed to adhere for 3 days and non-adherent cells were removed by replacing the medium. Upon reaching 80 ± 90% confluence, adherent pMSC were harvested by trypsinization and subcultured at 1:3 ratio in T75 flasks. Cells were cultured until they reached ∼ 80% confluence in each passage and collected for transplantation after 3–5 passages. The MSCs-like phenotype of the obtained cells was confirmed by flow cytometry (Figure 2). Mycoplasma contamination was checked by PCR. Before injection, pMSC were dissociated using Accutase^®^ (StemCells Technology, Canada). After dissociation, Accutase^®^ was inactivated with 100% knockout medium and the cell suspension centrifuged at 400 × *g* for 4 min. Prior to transplantation cells were washed twice with DPBS and counted. A dose of 5 × 10^5^ cells in 2 ml of saline was prepared for each intra-arterial transplantation.

### Flow Cytometry Analysis

Placental MSC and adult drNPC were washed with PBS containing 1% FBS. FITC-conjugated anti-human CD34, CD45, and CD105 antibodies and P-conjugated anti-human CD29, CD73 and CD90 antibodies were used for MSC staining. drNPC were stained with FITC-conjugated anti-human Nestin, b-III-tub, MAP2 and PE-conjugated anti human SOX2. All antibodies were purchased from Miltenyi Biotec. The analysis was performed with a CyFlow *Space* flow cytometer (Sysmex *Partec)* using the Partec FloMax^®^ flow cytometry Data Acquisition and Analysis Software.

### Cell Differentiation

The neurogenic differentiation potential of drNPC was determined by culture in the neuron-induction medium comprising Neurobasal (Gibco, United States), L-glutamine (1X, Gibco), antibiotics (1X, Gibco) and the growth factors BDNF 20 ng/ml and GDNF 20 ng/ml (both from Miltenyi Biotec). The osteogenic, adipogenic and chondrogenic differentiation potential of MSC was determined by culture in an osteogenic medium [DMEM supplemented with 10^–8^ M dexamethasone (Sigma, D4902), 10 mM b-glycerophosphate (Sigma, G9422), and 50 μg/ml ascorbic acid], adipogenic medium [DMEM supplemented with 10 mM 3 isobutyl-1-methylxanthine (Sigma, 17018), 0.1 mM indomethacin (Sigma, 17378), 10 μg/ml insulin (Sigma, I6634), 10^–6^ dexamethasone] or chondrogenic medium (Stempro, Invitrogen), respectively, with subsequent verification by staining with Alizarin Red (Sigma), Oil Red O (Sigma) or Alcian Blue (Sigma), respectively. A Nikon Eclipse Ci microscope (Nikon Instruments Inc, Japan) was used for image capture.

### Cell Labeling

Cells were labeled with superparamagnetic iron oxide (SPIO) microparticles (MC03F Bangs Laboratories, mean diameter 0.50 ± 0.99 μm) carrying Dragon Green fluorescent dye (λex = 480 nm, λem = 520 nm) and with lipophilic membrane red fluorescent dye PKH26 (Sigma-Aldrich) as previously described (Namestnikova et al., 2017b). Double cell labeling had no influence on cell viability (data presented in the Supplementary Material) and cell proliferation, as shown previously (Namestnikova et al., 2017b).

Determination of the average iron content per cell was carried out by inductively coupled plasma (ICP) atomic emission spectrometry (Agilent 4200 MP-AES, United States) using a calibration curve. Standard solutions with Fe (III) concentrations of 500, 1,000, 1,500, and 2,000 ppb were prepared by dilution of iron standard solution in 2% (w/w) HNO_3_ for ICP (Merck, United States). 2% aqueous solution of HNO_3_ was used as a blank. Samples were prepared by pelleting 2.4 × 10^6^ SPIO-labeled pMSC or drNPC by centrifugation and dissolving the pellet with 500 μl of concentrated nitric acid (stirring at room temperature, 48 h). Three independent measurements were carried out and the average iron content in cells was calculated as the mean of 3 experiments ± standard deviation.

### Animals

Healthy adult male Wistar rats weighing 250–300 g were purchased from AlCondi, Ltd., Moscow, Russia. Males were chosen to avoid the potential neuroprotective action of estrogens (Sacks et al., 2018). Animals were allowed to acclimate for 10 days before the experiment. Rats were housed in groups of four to five animals per plastic cage before surgery and individually after the MCAO. They were kept in standard conditions – 12-h/12-h light/dark cycle, temperature 22 ± 2°C and free access to standard rodent chow and water *ad libitum*. All surgical procedures and MRI studies were conducted under inhalation anesthesia (Aerrane, Baxter HealthCare Corporation, United States) supplied by the animal anesthesia system (E-Z-7000 Classic System, E-Z-Anesthesia^®^ Systems, United States): 3.5–4% isoflurane mix with atmospheric air for the induction of anesthesia and 2-2.5% isoflurane/air mix for its maintenance. Body temperature was kept around 37°C with a heating pad to prevent hypothermia during surgery and MRI. During all experimental stages laboratory rats were kept in comfort facilities, excluding influence of pain and the other negative factors. The researchers provided the control of the conditions of animals aimed to reveal the distinguishing features of pain, suffer or distress in rodents. In case of detection of signs of pain or distress, the analgesic (meloxicam) were administered to relieve them. For euthanasia at the end of the experiment and for histological studies animals were placed in the induction chamber (E-Z-7000 Classic System, E-Z-Anesthesia^®^ Systems, United States) and inhalation anesthesia were performed with a lethal dose of isoflurane. Afterward just before the transcardial perfusion animals were additionally injected with the lethal dose of Zoletil.

### Study Design

All rats recruited into the study were subjected to transient MCAO surgery (*n* = 70, 8 of which were excluded from the experiment due to unsuccessful surgery). 24 h after the MCAO the remaining 62 rats were allocated to one of the following groups: (1) control group including animals getting intra-arterial (IA) administration of 2 ml of saline (*n* = 21); (2) group of rats receiving IA administration of 7 × 10^5^ drNPC in 2 ml of saline (*n* = 18: 8 receiving labeled cells + 10 receiving unlabeled cells; and (3) group including rats receiving IA administration of 5 × 10^5^ pMSC in 2 ml of saline (*n* = 23, 9 receiving labeled cells + 14 receiving unlabeled cells).

Though the optimal time after stroke for the IA transplantation of cells has never been firmly established (Guzman et al., 2018; Baker et al., 2019; Fernández-Susavila et al., 2019) available data still allowed us to choose 24 h time window based on the following: (1) published data concerning the correlation between therapeutic time window and the efficacy of cell therapy in the acute phase after MCAO (Toyoshima et al., 2015); (2) commonly recognized increase of the blood brain barrier (BBB) permeability within 6–48 h after stroke onset (Merali et al., 2017); and (3) duration of the therapeutic window for current clinical reperfusion methods (systemic thrombolysis and endovascular thrombectomy) (Powers et al., 2019).

The dose of transplanted cells was the highest dose warranting safe IA administration without cerebral embolism. The dose of 5 × 10^5^ pMSC (average cell diameter 24–25 μm) was established in our previous study (Namestnikova et al., 2017a). The 7 × 10^5^ dose for drNPC was slightly higher, since drNPC have smaller diameter (average cell diameter 13–15 μm). The injection volume of 2 ml was also chosen based on our previous research (Namestnikova et al., 2017a) and analysis of the literature data (concise review (Guzman et al., 2018). No immunosuppressive treatment was performed.

To locate transplanted cells after their first and the following passages through brain vessels real time MRI examination with subsequent histological verification rats were injected with double-labeled cells.

Despite the fact, that the chosen double cell labeling had no influence on cell viability *in vitro*, unlabeled cells were used for evaluation of the therapeutic effects after drNPC and pMSC transplantation in order to reduce any unwanted variables in the study. Therapeutic efficacy was assessed based on analysis of survivalrate of animals, neurological deficit according to the modified neurological severity score scale (mNSS), infarct volume and hemorrhagic transformation of the ischemic zone calculated using MRI scans estimated just before and on the 7th and 14th day after cell transplantation. All tests were conducted by observers blinded with regard to the treatment groups.

Sample size assessment was made based on our previous study of the therapeutic effect (mNSS) of IA administration of iPSC-derived neural precursor cells into MCAO rats (Namestnikova et al., 2019). In that study the effect size of *f* = 2.64 was determined for two groups and three repeated measurements using Repeated-Measures ANOVA approach. In the current study, sample size was initially assessed on the assumption of power = 0.8 and significance level *p* = 0.05 for three groups with three measurements and the number of rats in each group should be minimum *n* = 6. Eventually, with regard to the high mortality rate within the first week after MCAO and the necessity to sacrifice rats for histological examination, the total number of rats subjected to MCAO was 70.

### Transient Middle Cerebral Artery Occlusion Model

Transient 90 min middle cerebral artery occlusion (MCAO) in rats was performed as described previously (Gubskiy et al., 2017). Briefly, the bifurcation of the right common carotid artery (CCA), the internal carotid artery (ICA) and the external carotid artery (ECA) were exposed under isoflurane anesthesia supplemented with subcutaneous injection of 0.1 ml of 0.5% bupivacaine at the ventral neck midline and intraperitoneal premedication with atropine sulfate 0.05 mg/kg in 1 ml 0.9% NaCl. After subsequent ligation of the ECA and pterygopalatine artery (PPA), microclips placement on the CCA and ICA, and electrocoagulation of the superior thyroid artery and the occipital artery, the ECA was cut with microscissors between two sutures. A silicon rubber-coated size 4-0 monofilament (Doccol Corporation, diameter 0.19 mm, length 30 mm; diameter with coating 0.37 ± 0.02 mm; coating length 3–4 mm) was inserted into the stump of the ECA and guided into the lumen of ICA for 18–20 mm toward the middle cerebral artery (MCA) until mild resistance was felt. The filament was fixed with 5-0 silk suture around the ECA stump and the microclip from the CCA was removed to restore blood flow. MCAO started at this moment and continued for 90 min. Immediately after the start of MCAO the surgical wound was closed and the animal was transferred into the MRI scanner for the control of filament position and checking for possible hemorrhagic complications. Ten minutes before the end of the occlusion period, rats were re-anesthetized and the incision reopened. The monofilament was slowly withdrawn from ICA exactly at the 90th minute after MCAO onset. The operation wound was rinsed with sterile saline and closed, 3 ml of sterile saline was injected intraperitoneally and 30 mg/kg gentamicin sulfate was given intramuscularly. In the case of successful filament placement with complete occlusion of the right MCA confirmed by MRI, the rat was placed in a preheated cage for recovery from anesthesia. Rats with hemorrhagic complications were excluded from the experiment. The rate of unsuccessful MCAO stroke modeling was 13% of (62 rats out of 70 were operated successfully).

### Cell Transplantation

Cell transplantation was performed under isoflurane anesthesia 24 h after MCAO. The right CCA, the ECA stump and the ICA were exposed, PPA was again ligated by a 5 ± 0 silk suture and microsurgical clips were placed on CCA and ICA. A microcatheter (rodent tail vein catheter with diameter 1F, 28 cm in case of the unlabeled cell administration or 1F, 90 cm MRI applicable catheter for SPIO labeled cell injection inside the MRI scanner, Braintree Scientific, Inc, INC, United States) filled with saline to prevent air bubbles was inserted into the stump of the ECA and advanced into the ICA for 5–6 mm from the bifurcation of CCA. The microsurgical clip on the CCA was removed to maintain the blood flow in the ICA during transplantation. At this stage, rats assigned for labeled cell transplantation were transferred to the MRI scanner for real-time MRI. In all cases the catheter was then connected to a 2 ml syringe placed in the microinjector and 2 ml of pMSC or drNPC suspension in saline, or just 2 ml of saline (control group) was delivered into the ICA over a 20-min time period. The catheter external diameter was small enough to allow blood flow around it during transplantation, which is crucial to ensure successful administration. After transplantation the catheter was removed, the ECA stump was electrocoagulated, and the incision was closed with a 5 ± 0 silk suture. The viability of cells after passing through the catheter was assessed in our preliminary experiments using an automated cell counter (Invitrogen), and its reduction proved to be insignificant (6–8%).

### Evaluation of the Therapeutic Effect

The assessment of therapeutic efficacy of the cell therapy was based on the survival rate and the modified neurological severity score (mNSS). The mNSS scale reveals motor, sensory, balance and other neurological disorders (Schaar et al., 2010; Balyabin et al., 2016). In each test one point is given for failure and no points for success. Motor function in post-operative rats was evaluated by the animal’s ability to move its head and limbs contralateral to the stroke side and walk straight. Visual, tactile, sensory and proprioceptive function testing was carried out. The ability to balance on a beam for at least 20 s was also estimated. In addition, mNSS takes into account the presence of neurological dysfunctions such as seizures, myoclonus, and myodystony. The maximum number of 18 points on the scale corresponds to the most severe neurological deficit. In this study all animals were blinded before the first testing. Assessment of the survival rate started at the time of transplantation, while the neurological status based on the mNSS scale was estimated just before and on the 7th and 14th day after the intra-arterial administration of cells or saline. Only after all data had been obtained and recorded, and all groups independently analyzed, were the results unblinded.

### Magnetic Resonance Imaging

The magnetic resonance imaging (MRI) was carried out using a 7T ClinScan system for small animals (Bruker BioSpin, United States). During the procedure rats were maintained under isoflurane inhalation anesthesia. MRI guided MCAO for control of the filament position and possible complications was performed as described previously (Gubskiy et al., 2017). Just before IA injection, high resolution Susceptibility Weighted Imaging (SWI; 3D Gradient Echo with RF spoiling and flow compensation; TR/TE = 50/19.1 ms; flip angle = 15; averages = 1; spectral fat saturation; FOV = 30 mm × 20.6 mm; slice thickness = 0.5 mm; matrix size = 256 × 176) was performed, followed by dynamic real time visualization of transplanted cells carried out for 24 min starting at the moment of transplantation by T2^∗^ weighted imaging with time resolution 1:00 min (2D Gradient Echo pulse sequence with RF spoiling; TR/TE = 279/14 ms; flip angle = 30; averages = 1; FOV = 24 mm × 24 mm; slice thickness = 1 mm; matrix size = 173 × 192). Immediately after real time visualization, high resolution SWI was performed again for more sophisticated SPIO-labeled cell imaging. MRI evaluation of infarct volume and hemorrhagic transformation of ischemic stroke was done just before, and at 7 and 14 days, after IA administration of cells or saline. In this study the MRI protocol included the following pulse sequences: diffusion-weighted imaging (DWI) with mapping of the apparent diffusion coefficient (ADC) (GRE Echo-planar pulse sequence; TR/TE = 9000/33 ms; b factors = 0 and 1,000 s/mm^2^; diffusion directions = 6; averages = 3; spectral fat saturation; FOV = 30 mm × 19.5 mm; slice thickness = 1.0 mm; matrix size = 86 × 56), T2–weighted imaging (T2 WI; Turbo Spin Echo pulse sequence with restore magnetization pulse; turbo factor = 9; TR/TE = 4,000/46 ms; averages = 2; spectral fat saturation; FOV = 37 × 29.6 mm; slice thickness = 0.5 mm; matrix size = 320 × 256; respiratory gated) and high resolution Susceptibility Weighted Imaging.

### MRI Data Analysis

Quantitative MRI data analysis was performed using ImageJ software (Rasband, W.S., ImageJ, U. S. National Institutes of Health, Bethesda, Maryland, United States^1^, 1997–2015). Infarct area on each slice of T2WI were outlined manually using freehand selection tool in the ImageJ and drawing tablet by radiologist with more than 5-year experience in experimental and clinical stroke visualization. The volume of the brain infarction was measured by summation of areas measured on adjacent cross-sections using the following formula: V = (S1 + … + Sn) × (h + d), where S1,…,Sn is area measured on slice n, where h is the slice thickness and d is the interval between the slices. The hemorrhagic transformation was assessed using the using the Heidelberg Bleeding Classification (von Kummer et al., 2015) by a radiologist. Each rat was assigned a score depending on the anatomic description of intracranial hemorrhages. Afterward, for data analysis rats with score 0 and 1a, and with 1b and higher, were combined. Distribution of SPIO-labeled cells was evaluated manually analyzing real time T2^∗^WI and high resolution SWI before and after injection. Comparing this data, labeled cells were defined as hypointense spots appearing on images after IA injection.

### Immunocytochemistry and Microscopy of Tissue Sections

The expression of neural stem cell, neuronal cell and glial cell markers was determined by immunocytochemical analysis. The cells were fixed by adding 4% buffered formaldehyde solution containing 0.1% saponin. Primary antibodies to Nestin (R&D, 2 μg/ml), SOX2 (BD Biosciences, 5 μg/ml), βIII-tubulin (R&D, 2 μg/ml), GFAP (DAKO, 5 μg/ml), MAP2 (Sigma-Aldrich, 5 μg/ml) and NF-200 (Sigma-Aldrich, 5 μg/ml) were used together with Alexa Fluor 488 goat anti-mouse IgG (H + L) or Alexa Fluor 633 goat anti-rabbit IgG (H + L) secondary antibodies (all 1:400; Invitrogen, SA). The cell nuclei were labeled with Hoechst 33342 stain (Thermo Fisher Scientific). Immunofluorescence was analyzed using a Nikon A1 scanning laser confocal microscope (Nikon Co., Japan).

For immunohistochemical studies animals were sacrificed by the inhalation anesthesia with a lethal dose of isoflurane and additionally just before the transcardial perfusion with intraperitoneal injection of a lethal dose of chloral hydrate at 1, 24, 48, 72 h, 7 days and 14 day after the IA transplantation of cells or saline. Transcardial perfusion was performed using phosphate buffered saline (PBS, 0.1M, pH 7.4), followed by ice-cold 4% paraformaldehyde (PFA) in 0.1M PBS. The animals were decapitated, the brains removed from the skull, post-fixed at 4°C overnight in the same fixative, washed three times with PBS and cryoprotected in 30% sucrose solution. Coronal sections 40 um thick were cut using a cryostat microtome (Leica CM1900). The fluorescence of Dragon green SPIO and Pkh26 was used to select sections containing transplanted cells. For immunohistochemistry brain sections were blocked with 5% normal goat serum for 30 min at room temperature, and incubated with anti-Rat Blood-Brain Barrier antibody (1:100, Biolegend^®^) or anti-Mitochondria antibody (1:100, Abcam^®^) diluted in a special solution (5% normal goat serum (Sigma-Aldrich^®^), 0.3% Triton x-100 (Triton^®^) and 0.01 M PBS (pH 7.4)) at 4°C overnight. Then sections were rinsed 3 times for 10 min with PBS followed by incubation with secondary antibodies (1:500, anti-mouse IgG Alexa fluor^®^ 647) for 2 h at room temperature. The nuclei were counterstained by incubating for 10 min at room temperature with DAPI solution (2 μg/mL, Sigma). Sections were mounted in 80% glycerol. For Perls’ Prussian blue staining the specimens were immersed in a mixture of 2% potassium ferrocyanide and 2% HCl for 10 min, rinsed with deionized water and counterstained with Neutral Red for 3 min. Fluorescence confocal micrographs were captured with the Nikon A1R MP + laser scanning confocal microscope. Bright-field images were acquired using the Keyence BZ9000E microscope.

### Statistical Analysis

The R software (“WebPower” library) was used for the sample size calculation (Cohen, 1988). Statistical analysis was performed using IBM SPSS Statistics 23 and R 3.5.1. *P* value <0.05 was considered significant. Results were expressed as the mean with standard deviation (SD). Animal survival was assessed by the Kaplan-Meier method using log-rank test (with FDR correction) for pairwise comparison of groups. For estimation of mNSS and infarct volume, comparisons between groups were performed using the General Linear Model with repeated measurements (the dynamics of changes within 14 days and differences of confidence intervals between groups on days 7 and 14 were evaluated; values were normalized based on the data captured on day 1). For the assessment of hemorrhagic transformation, two-sided Fisher’s exact test was performed.

## Results

### Characterization of drNPC and pMSC

Prior to transplantation, cell cultures were subjected to immunocytochemical and flow cytometric analysis. In the initial drNPC cultures both immunocytochemistry and flow cytometry revealed high levels of expression of the neural stem cell marker SOX2, neural marker βIII-tubulin, and astroglial marker GFAP (Figures 1A,B,E), while drNPCs maintained in the neuron differentiation medium expressed MAP2 and NF200, but did not express SOX2 (Figures 1C,D). The mesenchymal identity was confirmed by their potential for chondrogenic, osteogenic and adipogenic differentiation (Figures 2A–C). The CD profile of pMSC revealed by flow cytometry (CD34-, CD45-, CD105+, CD29+, CD73+, CD90+) was typical for MSC (Figure 2D). Both drNPC and pMSC expressed low levels of HLA-DR (major histocompatibility complex class II antigen), therefore immunosuppression was not carried out (Figures 1E, 2D).

### Iron Content per Cell

The average iron content determined by plasma-atomic emission spectrometry was 3.52 ± 0.37 and 3.32 ± 0.39 pg Fe/cell for labeled pMSC and drNSC, respectively. Thus, both types of stem cells incorporated almost equal iron load and therefore had similar magnetic properties on MRI. In a previous publication we reported on MRI visualization of single SPIO-labeled pMSC (average iron content 3.8 ± 0.3 pg/cell) in live rat brain (Namestnikova et al., 2017b). In the current study we used cells with similar iron load and the same MRI scanner, and, therefore, could likewise track single transplanted cells *in vivo*. Additionally, MRI data were verified by immunohistochemical analysis.

### DrNPC and pMSC Distribution in the Live Rat Brain

On MR-imagesSPIO-labeled drNPC and pMSC appeared as hypointense spots on T2^∗^ WI and SWI. As shown elsewhere (Namestnikova et al., 2017b), the above mentioned pulse sequences are the most sensitive for *in vivo* detection of small groups of labeled cells or even single cells. SPIO-labeled cells can be distinguished from hemorrhagic transformation of brain infarct and cerebral blood vessels by analyzing their shape on the series of adjacent slices and time of their appearance. In this study MRI data were verified by histological examination. Double cell labeling (SPIO-dragon green and PKH26) combined with immunohistochemistry (antibody against human mitochondria) allowed to match the hypointense spots on MR images with the histologically verified transplanted cell locations. Additionally, Perls staining and bright field microscopy were used to locate iron accumulation.

### First-Pass Distribution of drNPC and pMSC

**FIGURE 3:**
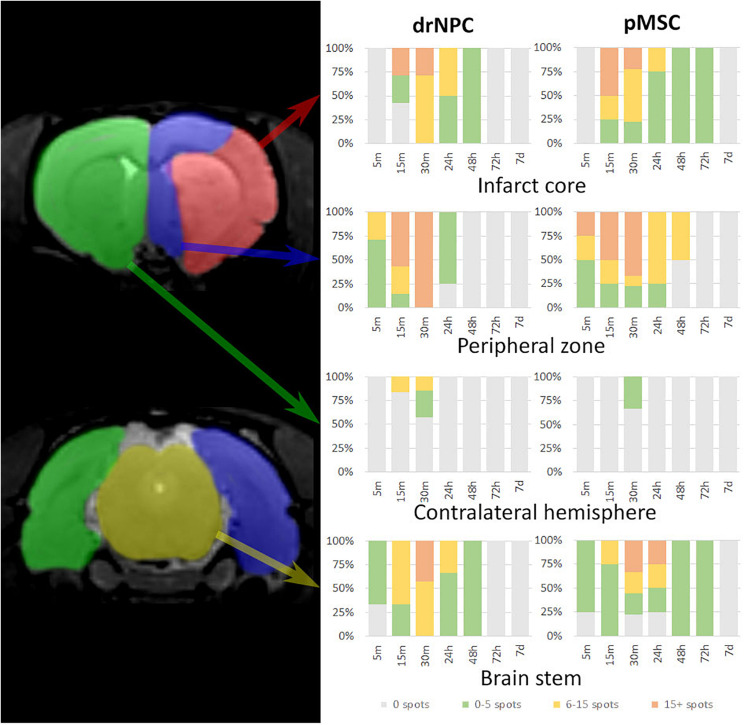
Diagrams of labeled drNPC and pMSC distribution during the entire observation period. The colors on the diagram indicate the number of hypointensive spots on SWI (zones of SPIO-labeled cell presence) in a particular area of the rat brain. The areas are schematically marked on the left images. Cell accumulation inside cerebral blood vessels started from 5 min onward after start of IA infusion. Neither drNPC nor pMSC seemed to undergo long-term homing: no drNPCs were observed within the brain more than 48 h after transplantation and no pMSCs could be seen after 72 h.

Figure 3 illustrates the overall distribution of transplanted drNPC and pMSC in the rat brain after MCAO and its changes with time, prepared on the basis of the real-time MRI data. Videos of the real-time MRI-visualization can be found in the Supplementary Material. Essentially, drNPC and pMSC were distributed in a similar way. Within the first 5 min, labeled cells were detected only in the peripheral zone of the infarct core and in the brain stem. After 15 min they entered the infarct core and could be found in the contralateral hemisphere. After 30 min the numbers of labeled cells trapped in all the described zones of the right (infarcted) hemisphere, especially in the peripheral region of brain infarct, reached their maximum. There were only minor differences between the real-time distribution of the two cell types, with the drNPC observed earlier and in greater numbers in the contralateral hemisphere than pMSC.

### Dynamical Distribution of drNPC and pMSC

**FIGURE 4:**
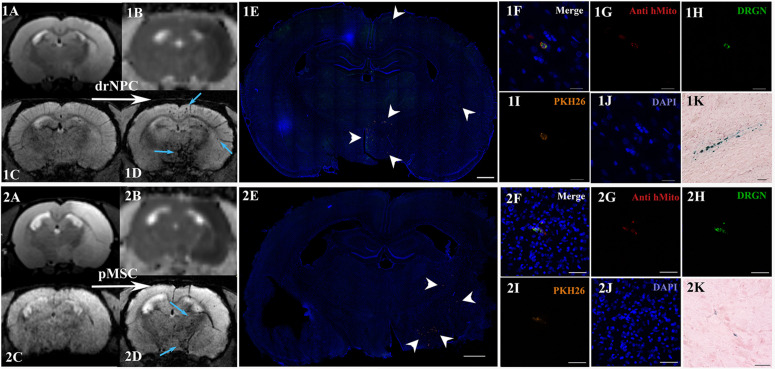
Distribution of drNPC and pMSC in the rat brain 30 min after IA transplantation. MRI and histological images of the rat brain before and after drNPC (top panel row) and pMSC (bottom panel row) infusion. **(1A,2A)** T2 WI of the rat brain before transplantation; the hyperintense zone corresponds to ischemic brain injury. **(1C,2C)** SWI of the rat brain before cell transplantation. **(1B,2B)** ADC maps (calculated from DWI) 30 min after cell transplantation; no new zone of cytotoxic edema and therefore no embolic infarction were detected. **(1D,2D)** SWI of rat brain 30 min after cell transplantation, blue arrows indicate hypointense zones of SPIO-labeled cell accumulation. Panoramic confocal fluorescence images of the coronal sections cut through the rat’s forebrain 30 min after transplantation of drNPC **(1E)** or pMSC **(2E)**. White arrowheads indicate double-labeled cells in the ischemic core and to the periphery of it. Scale bars: 1,000 μm. **(1F–1J,2F–2J)** High-magnification confocal fluorescent images of transplanted cells inside cerebral blood vessels. To validate the MRI data and check its consistency with the microscopy results, cells were double-labeled with the membrane dye PKH26 (orange), and SPIO microparticles fluorescent marker Dragon Green (green, present in the cytoplasm). Transplanted human cells were also stained using antibodies against human mitochondria (red). The scale bars: 20 μm for panels **(1F–1J)** and 50 μm for panels **(2F–2J)**. **(1K,2K)** Bright-field microscopy of Perls’ Prussian Blue stained sections showing SPIO accumulation (blue) in the brain. Scale bar: 50 μm.

**FIGURE 5:**
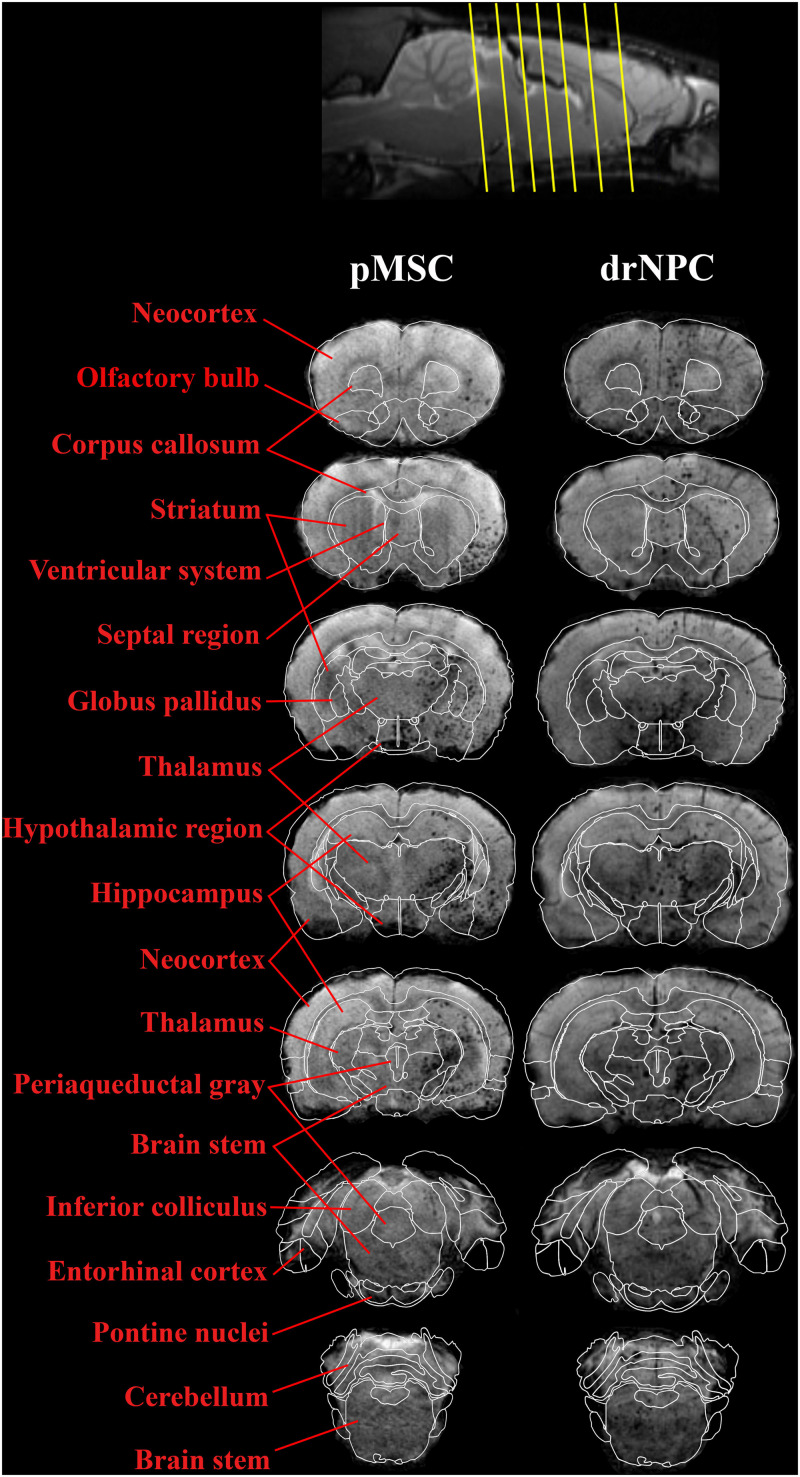
Overall distribution of drNPC and pMSC in the rat brain in relation to the anatomical structures. SWI MRI 30 min after IA injection of drNPC (the right column) and pMSCs (the left column), 7 coronal sections (levels are marked with yellow lines on the top). SPIO labeled cells are hypointense (dark) spots on SWI. Brain structures were signed in red and depicted with white lines on the base of “the Scalable Brain Atlas” (Papp et al., 2014; Bakker et al., 2015; Sergejeva et al., 2015).

**FIGURE 6:**
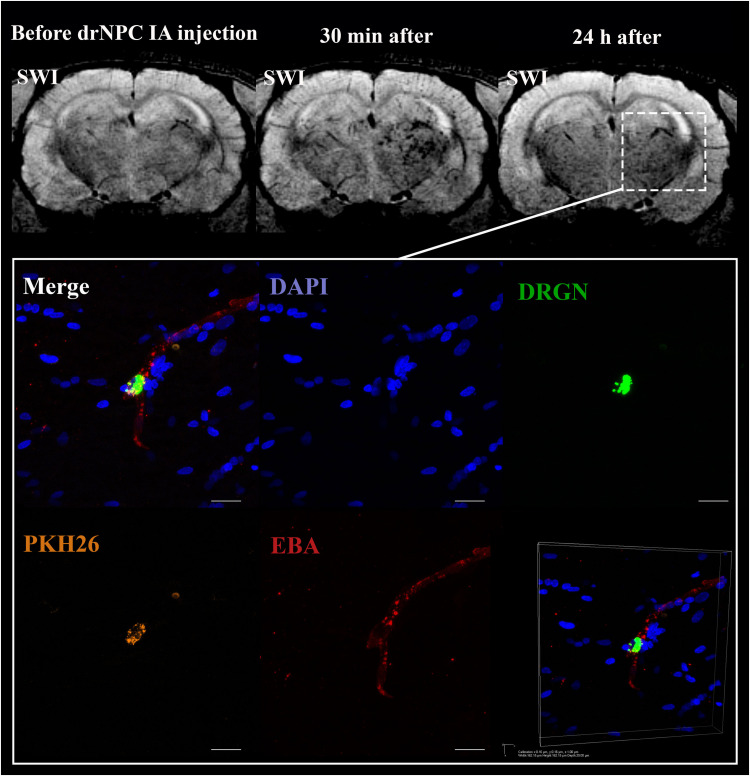
Location of drNPC inside the cerebral vessels after IA infusion. Top panel represents the SWI of the rat brain before and 30 min and 24 h after transplantation. White rectangle indicates the area of SPIO-labeled cell accumulation. Bottom panel demonstrates high-magnification confocal fluorescence microscopy images of double-labeled drNPC (PKH26 – orange, SPIO microparticles in the cytoplasm – green) inside the cerebral blood vessels (stained with EBA, red). Scale bars: 20 μm. Bottom right insert: 3D-reconstruction of z-stacks demonstrated the cells’ localization inside vessels.

**FIGURE 7:**
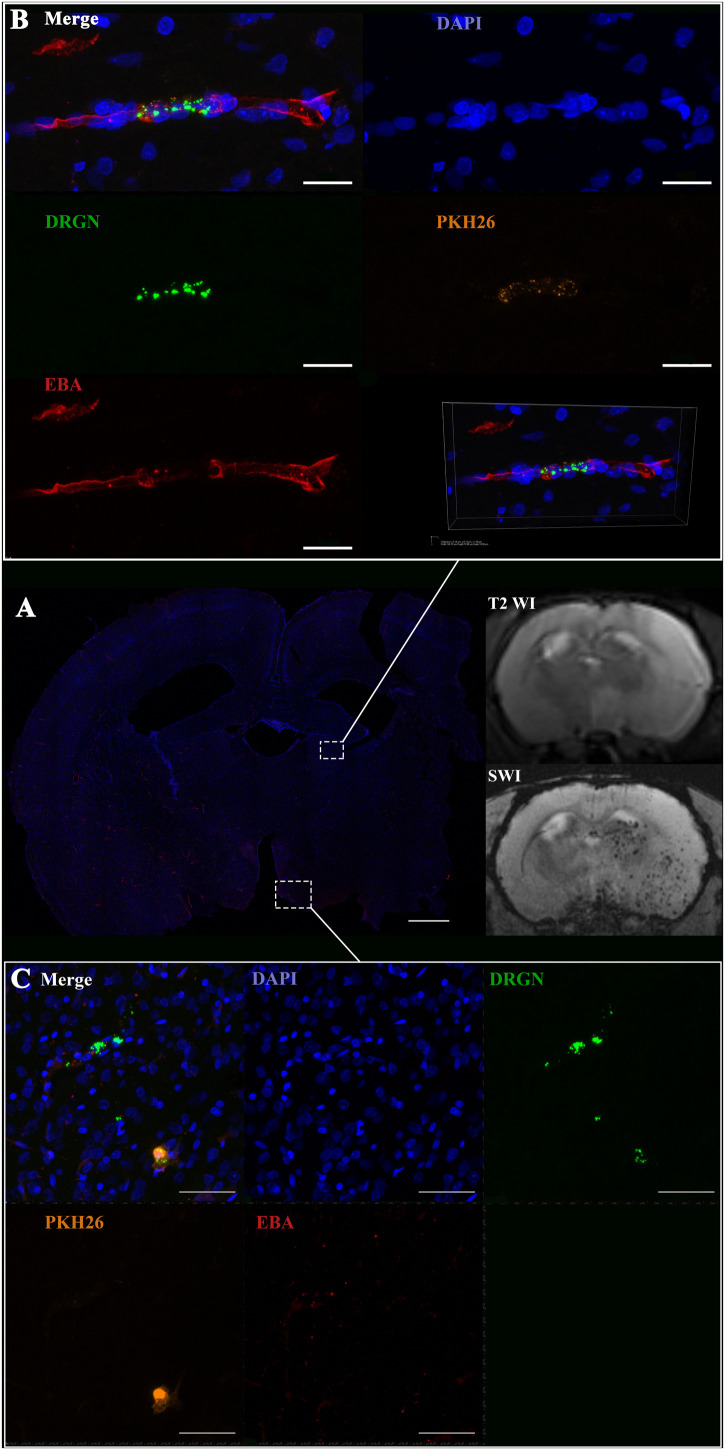
Location of pMSC in cerebral vessels after IA infusion. **(A)** Confocal fluorescence and MR-images of the coronal section of the rat brain 24 h after transplantation. Brain infarct zone is hyperintense on T2 WI, SPIO-labeled cells are hypointense on SWI. White rectangle indicates the areas of labeled cell accumulation inside blood vessels (EBA, red) in different parts of the brain. Scale bar: 1,000 μm. **(B)** High-magnification confocal fluorescence microscopy images from the zone peripheral to the infarct core. Double-labeled pMSC (PKH26 is orange, SPIO microparticles in the cytoplasm are green) are located inside cerebral blood vessels, immunostaining for endothelial barrier antigen (EBA, red) is strong. Scale bars: 20 μm. **(C)** High-magnification confocal fluorescence microscopy images from the infarct core. EBA staining is weak, pMSC are in contact with the vessels and probably could pass into the brain parenchyma. It is worth noting, that the both double labeled cells and several cells with only single SPIO label can visualized. In our experiments such phenomena were detected in rare cases. Scale bars: 50 μm.

**FIGURE 8:**
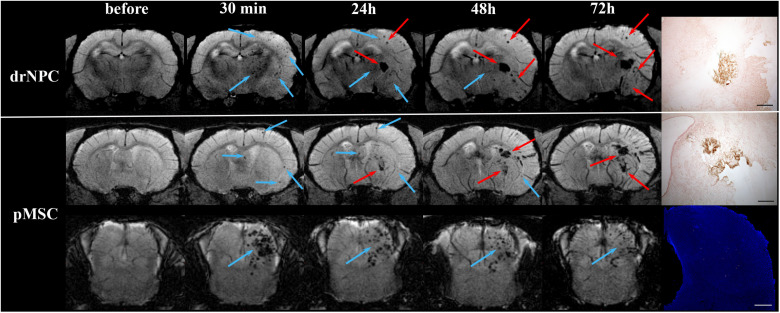
Dynamic distribution of drNPC and pMSC in the rat brain. SWI MR-images before and 30 min, 24 h, 48 h, 72 h after transplantation. Upper row: SWI coronal slices at the level of the forebrain after drNPC infusion. Bottom row: SWI coronal slices at the level of the forebrain and brain stem. Blue arrows indicate SPIO-labeled cell accumulation. drNPC persist in the brain up to 48 h; pMSC a little longer, up to 72 h. MRI data was verified by confocal microscopy (bottom row, scale bars: 500 μm). Red arrows indicate hemorrhagic transformations of ischemic stroke. Intracerebral microhemorrhages started to appear 48 h after MCAO (24 h after cells injection) and then increased. SPIO-labeled cells can be distinguished from hemorrhagic transformations and also cerebral vessels by analyzing their shape and time of appearance. Zones of hemorrhagic transformations confirmed by brightfield microscopy (Perl’s blue staining). Scale bars: 500 μm.

The subsequent fate of IA transplanted drNPC and pMSC in the brain are presented in Figures 4–8. Figure 4 demonstrates MRI and histological images of the rat brain 30 min after IA injection. It is worth noting, that IA transplantation of either drNPC and pMSC with adjustment parameters caused no microembolism of cerebral arteries and no thromboembolic stroke formation, which was confirmed by DWI (Figures 4,1B,2B). Hypointense spots on SWI shown with blue arrows (Figures 4,1D,2D) point to the sites of SPIO-labeled cell locations in one coronal brain section. MRI data were verified by histological examination, which demonstrated double-labeled drNPC or pMSC accumulation in the blood vessels in the brain infarct and peripheral to it in thalamic and hypothalamic regions. By analyzing biodistribution of each transplanted cell type, we found correspondence between the cell locations and anatomical structures of the brain. Figure 5 clearly illustrates that the distribution of drNPC and pMSC throughout the brain was almost identical. Labeled cells were found in the motor and sensory neocortex, corpus callosum, periventricular zone, brain stem and cerebellum, but mostly in the thalamus, striatum, hypothalamus and hippocampus. Single cells were also visualized in the contralateral hemisphere, more often in the neocortex of the frontal lobe and the rostral part of the striatum.

Curiously, despite a number of reports claiming penetration of systemically transplanted cells into the damaged brain (see section “Discussion”), in this study neither drNPC nor pMSC seemed to actively invade brain tissue. Immunostaining for the endothelial barrier antigen (EBA) demonstrated that in many brain regions the majority of injected drNPC (Figure 6) and pMSC (Figure 7) remained localized inside cerebral blood vessels in contact with the walls, in some cases possibly infiltrating the vessel walls. As seen at Figure 7C, the EBA staining was weak in the infarct core, likely reflecting the ischemia-related disruption in the blood-brain barrier. Considering all this, it cannot be excluded that single cells can pass through the blood brain barrier into the brain parenchyma in the infarct zone.

Over the 2–3 day period after IA transplantation, the number of drNPC or pMSC detected by MRI in the brain rapidly decreased (see Figure 8). At first transplanted cells could no longer be detected in the contralateral hemisphere, and then later no longer in the peripheral zone of ischemic lesion. In the infarct core and the brain stem transplanted cells persisted for up to 48 h in the case of drNPC and up to 72 h in the case of pMSC (see Figures 3, 8). Thus, neither drNPC nor pMSC seemed to undergo long-term homing.

### Comparative Therapeutic Efficacy of drNPC and pMSC Transplantation

#### Survival Rate

**FIGURE 9:**
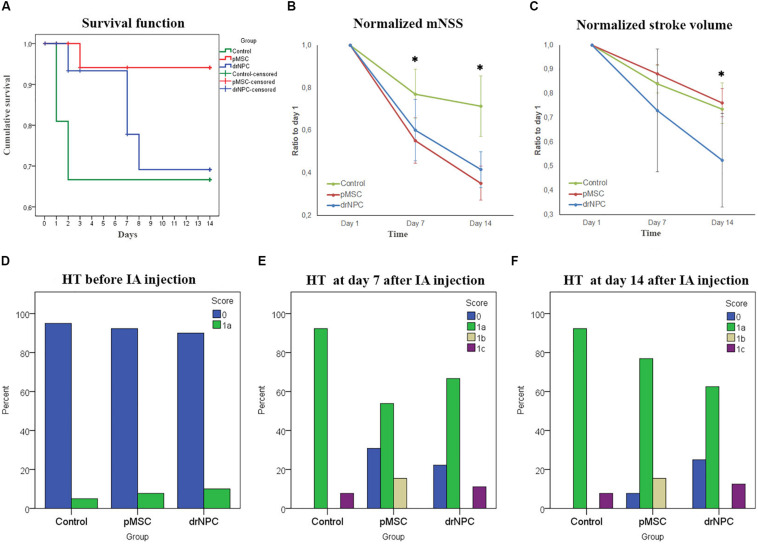
Therapeutic effects of cell transplantation and estimation of hemorrhagic transformation after cerebral infarction. **(A)** Kaplan—Meier survival curves for rats of the control group (green line), drNPC transplantation group (blue line), and pMSC transplantation group (red line) over 14 days observation period. Statistical analysis was performed by log-rank test and revealed significant improvement (*p* < 0.05) in animal survival rate in the pMSC group in comparison with the other two groups. **(B)** Neurological deficit was estimated using mNSS before and at 7 and 14 days after IA administration of saline (green line), drNPC (blue line) or pMSC (red line). The dynamics of changes of mNSS score in animals after transplantation of drNPC or pMSC significantly differ from that in the control group: the animals in groups with transplantation of either cell type had significantly lower mNSS scores compared to the control group at 7 and 14 day. **(C)** Stroke volume was evaluated using MRI performed just before and on days 7 and 14 days after IA administration of cells or saline. The dynamics of changes in stroke volume in the drNPC group significantly differed compared to the pMSC and control groups. Administration of drNPC caused a significant decrease of stroke volume by the end of the second week after transplantation. Data at graphs B-C are normalized to day 1 and expressed as mean ± SD, *asterisk indicates significant differences of *p* < 0.05. **(D–F)** Hemorrhagic transformation (HT) of brain infarction after IA injection of drNPC, pMSC or saline. Data are given according to the Heidelberg scale. 0: no hemorrhage, 1a: single petechial bleedings, 1b: confluent petechial bleedings, 1c: hematoma within infarcted tissue, occupying <30% with no substantive mass effect. Frequency of hemorrhagic transformation of brain infarction in rats from all experimental groups was estimated and no significant differences were observed before **(D)**, on 7 **(E)** and 14 **(F)** days after IA injection.

Survival rates were monitored for 14 days after IA administration of saline, drNPC or pMSC. Kaplan-Meier survival curves are presented at Figure 9A. The pairwise comparison of experimental groups was performed by the log-rank test and showed that transplantation of pMSC significantly enhanced the survival of animals. At the same time the drNPC group showed a trend toward better survival, however, the differences were not statistically significant.

#### Neurological Outcomes

The neurological deficits developing in the rats subjected to MCAO were evaluated using mNSS over a 14 days period for three times: just before cell transplantation (i.e., 24 h after MCAO) and on the 7th and 14th day post-transplantation. Results are shown at Figure 9B. The dynamics of change in the neurological deficits in each treatment group differed significantly on both day 7 and 14 from the control group of rats that were injected with vehicle only (saline). The degree of neurological improvement was not significantly different between the drNPC and pMSC groups.

#### Infarct Volume

The volume of cerebral infarct is an important criteria shaping the clinical outcomes of ischemic stroke. In this study the infarct volume dynamics was estimated on MRI during a 14 days period using T2WI (Figure 9C). The dynamics of change of the infarct zone volume in animals after transplantation of drNCP significantly differed from that in the pMSCs and control groups. IA administration of drNPC caused a significant decrease of stroke volume by the end of the second week after transplantation, while IA infusion of pMSC had no effect. No significant differences between the pMSC transplantation group and the control group were observed.

#### Evaluation of Hemorrhagic Transformation of Ischemic Stroke

Hemorrhagic transformation is a common complication of ischemic stroke. In this study possible hemorrhagic transformation of the infarcted brain tissue was visualized by SWI. SWI was performed just before IA injection and immediately after it, and newly appearing hypointense spots on the MR-images were considered as labeled cells. Further examinations were performed in dynamics at days 7 and 14 after the IA administration. All newly appeared in the infarct core hypointense zones on SWI (in comparison with SWI at day 1) were considered as hemorrhagic transformations. The shape of spots and histological data were also taken into consideration. MR images were analyzed using the Heidelberg Bleeding Classification (von Kummer et al., 2015) generally accepted in clinical practice. At all time points the frequency of hemorrhagic transformation between all the groups did not significantly differ and the incidence of serious hemorrhagic complications remained at a low level (Figures 9D–F). Hence, in our hands IA transplantation of drNPC or pMSC did not increase the risk of hemorrhagic complications.

## Discussion

The main goal of this work was to study the first-pass and delayed distribution, as well as homing within the brain of human directly reprogrammed neural precursor cells and mesenchymal stem cells after IA transplantation in rats with MCAO stroke model. Additional goal was to evaluate the therapeutic effects of IA transplanted directly reprogrammed neural precursor cells in experimental stroke model and compare them to those of pMSC used as a positive control with proven efficacy.

### Safety of Transplantation

In the current study DWI started immediately after the drNPC or pMSC IA infusion and showed no thromboembolic stroke formation, thus confirming the safety of our cell infusion parameters. Many preclinical studies have shown IA cell transplantation aimed to deliver stem cells directly to the ischemic brain tissue is associated with potential risk of microembolism and formation of additional thromboembolic strokes. The problem was thoroughly examined in a review on the safety of IA cell transplantation in experimental stroke by Guzman et al. (2018), who identified several factors crucial for the transplantation outcome: cell type and size, cell dose, infusion speed, treatment window, and the extent of preservation of the natural arterial blood flow in the feeding vessel. The infusion parameters used in this study were selected with regard to these considerations and based on our previous work (Namestnikova et al., 2017a). Importantly, our past and present data confirmed the pivotal role of the maintenance of the arterial blood flow in the prevention of cerebral embolism.

Another safety issue we had to deal with was the possibility of the increased risk of intracerebral hemorrhages after drNPC or pMSC IA transplantation. Hemorrhagic transformation is a frequent spontaneous complication of ischemic stroke, that can be additionally provoked by different factors, such as thrombolytic therapy with the tissue plasminogen activator (Sussman et al., 2013). Transplantation of either drNPC or pMSC did not significantly increase the incidence of hemorrhagic transformation of the infarct zone.

As a matter of fact, the achieved consistency of the cell transplantation procedure and efficient real time monitoring of possible complications ensured that we truly studied the therapeutic effects of cell transplantation in experimental stroke unblurred by side effects.

### Biodistribution drNPCs and pMSCs in the Brain

In the current study the distribution of superparamagnetic iron oxide (SPIO) labeled drNPC and pMSC in live brain was explored during the whole period of their stay within the brain starting from the first-pass through cerebral vasculature. This was achieved by performing intra-arterial transplantation directly inside the MRI scanner. We followed the real time distribution of IA infused cells in the brain during infusion and immediately after it. Subsequently, the transplanted cells’ fate was assessed 24, 48, 72 h, 7 days and 14 days after infusion. In our study real time MRI allowed detection of the movement and distribution of single cells within the cerebral blood vessels during the infusion procedure. This approach was proposed by Walczak et al. (2017) who used GRE-EPI (gradient echo-echo planar imaging) pulse sequence with 2 s time resolution and low spatial resolution. We aimed to find a compromise between time and spatial resolution to achieve better image quality, and chose to use T2^∗^ gradient echo pulse sequence with time resolution of 60 s. This allowed more efficient visualization of labeled cells during intra-arterial infusion. Additionally, we controlled possible embolic events by using diffusion-weighted images after injection, as described above.

Dynamic visualization of transplanted cells revealed an almost identical pattern of real time distribution for both types of cells. Immediately after transplantation cells at first were observed in the peripheral zone of the infarct core and the brain stem, and only after ∼15 min were they detected in the infarct core and the contralateral hemisphere. Notably, in the contralateral hemisphere drNPC were seen earlier and in greater numbers than pMSC, likely due to their smaller size (average diameter 13–15 μm) compared to pMSC (24–25 μm). In our experiments the distribution of the transplanted cells in the ischemic area remained uneven during the transplantation time with substantially more cells remaining at the periphery. These results are partly in agreement with those of Walczak et al. (2017), who also performed real time MRI tracking of the IA transplanted cells in live brain after SPIO labeled MSC infusion through the ipsilateral ICA and demonstrated initial accumulation of cells in the peripheral stroke zone, followed by delayed cell inflow into the infarct core. However, no cells were observed in the contralateral hemisphere, and, therefore, the authors concluded that IA transplanted cells did not re-enter circulation and just had no chance to show up in the contralateral brain. Interestingly, our results do not totally contradict the notion of the cells not re-entering circulation after the first passage through brain vessels, since transplanted cells were detected in the rostral part of the contralateral hemisphere within anterior and azygos anterior cerebral artery vascular territory suggesting the overflowing of infused cells in the circle of Willis as a possible way of assessing the opposite half of the brain.

Factors determining the distribution of transplanted cells within the brain after IA transplantation have not been fully disclosed. Likely a significant, if not a crucial, contribution is made by cerebral perfusion (Walczak et al., 2017). Indeed, in this study and partially in some other works, the highest accumulation of cells after injection into the ICA of animals with experimental ischemic stroke was detected ipsilateral in the peri-infarct area (Oh et al., 2015), particularly in the thalamus, hypothalamus, striatum (Guzman et al., 2008), hippocampus (Guzman et al., 2008; Rosenblum et al., 2015). The reason of such distribution may be the cerebral vascular anatomy, i.e., the coexistence of arterial anastomotic networks stemming from different cerebral arteries in these brain regions (Paxinos, 2014). In addition, cell distribution is also affected by cell adhesion properties of the vascular wall varying in the brain region-dependent manner and chemotactic recruitment of transplanted cells by the chemokine-secreting damaged brain tissue (Ullah et al., 2019). For example, it was shown that the expression of vascular cell adhesion molecule-1 (VCAM-1) in the microvasculature of the ischemic area increases at day 1 with a gradual decline 3 days after stroke, and this overlapped with the distribution of the injected NPCs (Justicia et al., 2006; Guzman et al., 2008). Interestingly, in our experiments we observed rapid reduction of the drNPC and pMSC number within the first 2–3 days after administration.

We demonstrated that transiently homed transplanted cells, drNPC and pMSC alike, were localized inside blood vessels in close contact with the vascular wall. Though the passage of single cells through the BBB disrupted in the infarct core cannot be completely excluded, we did not observe this in the current study. Literature data concerning the fate of transplanted NPC and MSC are contradictory. Lundberg et al. (2012) found no detectable tissue engraftment of human NPC after IA transplantation, while others observed transendothelial migration of NPC into the brain parenchyma (Pendharkar et al., 2010; Horie et al., 2011; Doeppner et al., 2015; Rosenblum et al., 2015) and even neuronal/glial differentiation of transplanted cells (Guzman et al., 2008; Andres et al., 2011). The possibility of MSC transmigration across the endothelium is supported by the results of experiments using *in vitro* (Schmidt et al., 2006; Matsushita et al., 2011) and *ex vivo* (Al-Sowayan et al., 2019) models. Despite the *in vitro* evidence, it remains unclear whether IA injected MSC are able to cross the blood brain barrier or are passively captured at sites of severe brain injury and vessel disruption (Liu et al., 2013). In the initial studies some authors detected the accumulation of transplanted MSC at the site of stroke after systemic administration (Gutiérrez-Fernández et al., 2011; Kholodenko et al., 2012). Furthermore, Walczak et al. found that IA transplanted rat MSC were originally found inside blood vessels and then, in the stroke hemisphere, actively migrated across the vessel walls to the brain parenchyma (Walczak et al., 2008). It may be that the use of human cells in the current study have differences compared to rat cells used in a rat model of stroke. However, it has been also demonstrated using intravital microscopy, single-photon emission computed tomography and immunohistochemistry that after intracarotid administration, MSC just pause for a while within cerebral blood vessels, but do not transmigrate efficiently into the cerebral parenchyma (Cui et al., 2017). In recently published work of Andrzejewska et al. (2020) it was shown that human MSC after IA transplantation are capable of docking to the cerebral vessel walls and homing to the perivascular space. The authors demonstrated fast clearance from cerebral vessels of the majority of transplanted cells within the next 2 days after IA injection. In accordance with our data, at 72 h after cell infusion, transplanted MSC could be also only rarely found in the perivascular niches.

### Therapeutic Effects of Cell Transplantation

In the present study, both drNPC and pMSC showed therapeutic efficacy after IA transplantation in the rat MCAO model of ischemic stroke. However, some of their effects differed. Both cell types improved neurological function according to the mNSS by day 7, but statistically significant enhancement of the rate of the cerebral infarct volume reduction compared to the control group was observed only after drNPC transplantation, while pMSC showed no improvement on this parameter during the first 2 weeks after MCAO. In comparison, the influence of drNPC and pMSC on the survival rate after stroke followed a different pattern. There was significant increase of the survival rate compared to the control group in the pMSC group, while the drNPC group showed a trend toward better survival, the differences were not significant. The cause for these differences is a subject of further study, but we hypothesize that the greater anti-inflammatory effects of pMSC (Yarygin et al., 2016; Weiss and Dahlke, 2019) may have contributed to the better survival observed in the pMSC implanted group.

The therapeutic activity of the drNPC used in the present study have been previously demonstrated in another animal model of smaller focal ischemic stroke in mice by Vonderwalde et al. (2020). In that study 1 × 10^5^ drNPC were infused intracerebrally directly into the ischemic lesion in the sensorimotor cortex at 4 days post-stroke. Cell transplantation promoted functional recovery by day 32 of the experiment, but did not affect the ischemic lesion volume and the extent of gliosis. Here we describe a faster and more prominent positive therapeutic effect in a rat stroke model with a larger lesion in the MCA region. The observed differences with Vonderwalde et al. are probably the result of different stroke models, timing, and especially the choice of intra-arterial route of cell administration. It is noteworthy, that compared to intraparenchymal (IP) cell transplantation, IA transplantation is more convenient in terms of clinical translation. IP cell delivery provides accurate control of the graft placement, but involves intracranial manipulations that could be challenging in acute stroke patients (Kokaia and Darsalia, 2018; Baker et al., 2019). This still remains the case despite the results of recent clinical trials demonstrating relative safety of IP cell transplantation (Qiao et al., 2014; Kalladka et al., 2016; Steinberg et al., 2016). IA injection is less invasive and much more accessible in the era of endovascular thrombectomy (Guzman et al., 2018; Boltze and Jolkkonen, 2019).

A number of preclinical studies have demonstrated that neural stem/precursor cells (NSC/NPC) derived from the other sources (from adult or fetal brain tissues, embryonic stem cells, induced pluripotent stem cells) have also promoted functional recovery after intra-arterial transplantation into animal models of stroke (Andres et al., 2011; Doeppner et al., 2015; Rosenblum et al., 2015; Kondori et al., 2020). However, the extent of the positive effects have been somewhat different. Rosenblum et al. (2015) demonstrated that human embryonic stem cell-derived NSC pretreated with brain-derived neurotrophic factor (BDNF) increased sensorimotor recovery on day 14 post-transplantation, but failed to reduce the stroke volume compared to control animals. In another study Guzman et al. (2008) also found significantly better sensorimotor recovery but no significant difference in the stroke size after IA administration of mouse CD49d + NSCs at 17 days post-stroke. In contrast, Andres et al. (2011) revealed simultaneous decrease of infarct size and neurological deficit at day 3 post-stroke in mice receiving mouse postnatal C-C chemokine receptor 2 (CCR2 + / +) NSC. Similar results were reported by Kondori et al. (2020) after adult rat NSC transplantation. Though exact causes of the described differences are not clear, they are probably due to the variations of the properties of transplanted cells themselves that is related to their origin and culture conditions. Even NPC with similar phenotypes can have dissimilar functionality. For example, Lupatov et al. (2017) demonstrated that, despite the CD profile identity, the *in vitro* immunological properties of human stem/precursor cells dramatically changed depending on whether the culture medium contained FCS or a combination of growth factors commonly used to maintain NSC/NPC. Obviously, more studies are needed. However, the potential enhancement of functional neurological recovery after IA NSC/NPC transplantation is beyond doubt. Beneficial effects of cell therapy of experimental stroke with NSC/NPC delivered by IP administration are also quite evident.

Positive results have also been seen in the efficacy of MSC, although these results have varied more widely. Transplantation of MSC in animal models of stroke have been shown to attenuate neurological deficit, especially impairment of the motor function regardless of the cells delivery route (Vu et al., 2014; Wu et al., 2017; Sarmah et al., 2018; Zhang et al., 2018). Literature data about MSC therapy effects on the volume of ischemic lesions are more controversial. Some studies demonstrated decrease of the stroke volume evaluated by MRI or histologically (Yarygin et al., 2009; Komatsu et al., 2010; Ma et al., 2013; Yavagal et al., 2014). Other authors (Gutiérrez-Fernández et al., 2011; Watanabe and Yavagal, 2016; Zhang et al., 2018) found no influence of MSC transplantation on stroke volume. In this study we observed improvement of the survival rate and enhancement of neurological recovery after IA administration of pMSC, but did not find significantly faster reduction of the stroke volume during the first 2 weeks after MCAO compared to the vehicle control group. Again, as with transplantation of NSC/NPC in experimental stroke models, the reason for the inconsistency of results with regard to the infarct size changes is not clear. Most effects of MSC transplantation are commonly attributed to their immunomodulatory and anti-inflammatory activity (Wang et al., 2018; Dabrowska et al., 2019), but further specification of the concrete mechanisms is needed.

## Conclusion

In the present study we demonstrated real-time dynamical distribution of drNPC and pMSC in the rat brain after IA transplantation. We reveled the almost identical pattern of distribution for both transplanted cell types. During the first passing through cerebral circulation the majority of cells accumulated peripherally to the infarct core, in the subcortical regions and brain stem, and only after ∼15 min a smaller part of injected cells was detected in the infarct core and in the rostral part of the contralateral hemisphere We did not observe the transmigration of DrNPC and pMSC trough blood brain barrier to the brain parenchyma. Injected cells transiently homed inside the cerebral blood vessels close sticking to their walls for no longer than 2–3 days after administration.

Taken together, our results suggest that in the rat MCAO stroke model, IA transplanted drNPC and pMSC promote positive therapeutic effects by a paracrine mechanism, and possibly through cell-cell interactions and protective effects exerted in the brain perivascular spaces. Our data also indicate that the long-term survival of transplanted cells is not necessary for maintaining continued functional recovery. drNPC and pMSC may act through an indirect «trigger» mechanism launching a cascade of molecular and cellular events promoting recovery. The exact mechanism by which transplanted cells mediate their positive effects requires further studies.

## Data Availability Statement

The raw data supporting the conclusions of this article will be made available by the authors, without undue reservation.

## Ethics Statement

The studies involving human participants were reviewed and approved by the local Ethical Committee of the Pirogov Russian National Research Medical University (Protocol No. 140 from December 15, 2014). The human biological material was obtained from the Perinatal Center of Kama Children’s Medical Center (KCMC) of Naberezhnye Chelny. The studies of cell transplantation into human participants were not conducted. The patients/participants provided their written informed consent to participate in this study. The animal study was reviewed and approved by the local Ethical Committee of the Pirogov Russian National Research Medical University (Protocol No. 140 from December 15, 2014) and of Federal Research and Clinical Center of Specialized Medical Care and Medical Technologies (protocol No. 1_6_2019 from April 9, 2019). Experiments were carried out in accordance with directive 2010/63/EU on the protection of animals used for scientific purposes of the European Parliament and the Council of European Union dated September 22, 2010. All efforts were made to minimize the number of animals and exclude pain and other unpleasant effects for the animals. There were no restrictions in access to water and food (rodent chow), or other limitations. Animal studies are reported according to ARRIVE guidelines.

## Author Contributions

DN, IG, VBa, and KY contributed to conception and design of the study. DN and KY provide project administration. DN, IG, VR, KS, PM, AG, EC, DV, VK, VBu, AS, MA, and J-EA made investigations. LG, VC, VBa, and KY supervised the project. KY, VC, J-EA, and VBa provided Funding acquisition. DN and IG wrote the first draft of the manuscript. VR, KS, and AS wrote sections of the manuscript. DN, IG, KS, and KY edited the manuscript. DN and KS organized the database. IG performed the statistical analysis. All authors contributed to manuscript revision, read, and approved the submitted version.

## Conflict of Interest

The authors declare that the research was conducted in the absence of any commercial or financial relationships that could be construed as a potential conflict of interest.

## Footnotes

1 http://imagej.nih.gov/ij/
