# Supplementary material for: Intra-Arterial Stem Cell Transplantation in Experimental Stroke in Rats: Real-Time MR Visualization of Transplanted Cells Starting With Their First Pass Through the Brain With Regard to the Therapeutic Action
Source: Front Neurosci. 2021 Mar 2;15:641970. doi: 10.3389/fnins.2021.641970 (PMC7960930; doi:10.3389/fnins.2021.641970)
Supplement: Supplementary file 1 [file Table_1.DOCX]

Supplementary Material

Effects of double cell labeling on cell viability

Placental MSC and adult drNPC were labeled with superparamagnetic iron oxide (SPIO)-containing microspheres (MC03F Bangs Laboratories, mean diameter 0.50±0.99 μm) carrying Dragon Green fluorescent dye (λex = 480 nm, λem = 520 nm) and with PKH26 red fluorescent dye (Sigma-Aldrich). After 30 min in room temperature, two drops of NucRed™ Live 647 ReadyProbes™ Reagent (Thermo Fisher, USA) were added to the cell suspension. Cells were incubated with the reagent for 30 min. Before analysis cells were washed in DPBS twice. The analysis was performed with a CyFlow Space flow cytometer (Sysmex Partec) using the Partec FloMax® flow cytometry Data Acquisition and Analysis Software. The amount of cell death was less than 15%, indicating that double cell labeling did not significantly influence cell viability (data given on Supp. Fig. 1)


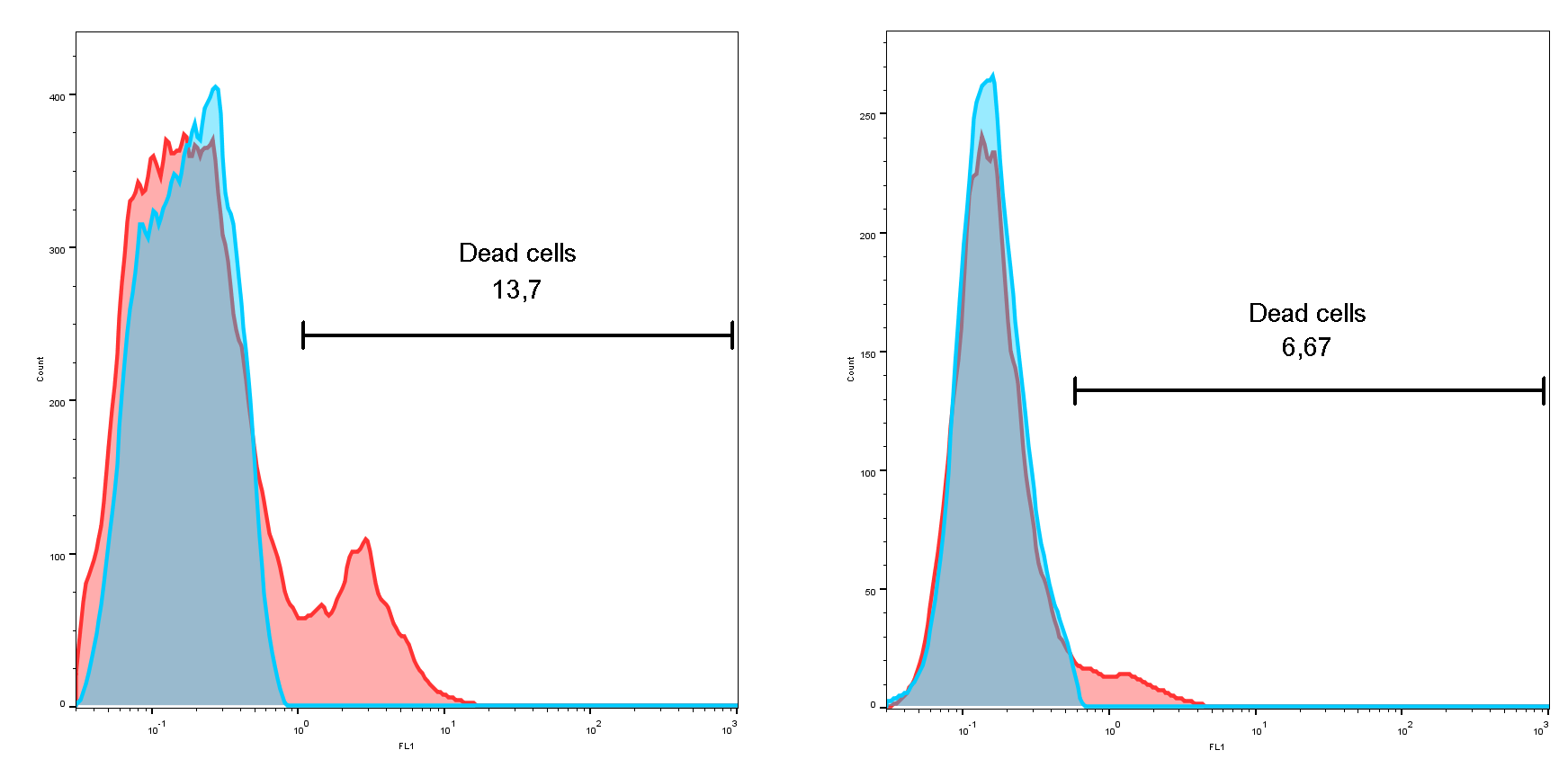


**Supplementary Figure 1.** Flow cytometry analysis of drNPC and pMSC viability with double cell labeling (SPIO + PKH26). Pink graphs is the negative control (isotype control immunoglobulins); blue graphs represent an experimental sample. The histograms show that the number of dead cells were less than 15%, thus double cell labeling had no significant influence on the cell viability.

Real time MRI of labeled drNPC and pMSC distribution in the rat brain (video captions)

A - Dynamic T2* WI with time resolution of 1 minute carried out for 24 minutes starting at the moment of cell infusion inside the MRI scanner. Zones of SPIO-labeled cell accumulation are hypointense (dark spots) on T2* WI.

B - T2 WI of the rat brain before cell injection, hyperintense zone in the right hemisphere corresponds to the infarct core;

C - DWI of the rat brain before cell infusion;

D - High resolution SWI performed immediately after real time visualization for more sophisticated SPIO-labeled cell imaging. SPIO labeled cells are hypointense spots on SWI;

E - DWI performed immediately after cell transplantation, no new zones of cytotoxic edema and therefore no thromboembolic lessons were detected compared to DWI before injection (C).
